# Differential Species Richness and Ecological Success of Epiphytes and Hemiepiphytes of Neotropical Araceae and Cyclanthaceae

**DOI:** 10.3390/plants12234004

**Published:** 2023-11-28

**Authors:** Erin C. Riordan, Katharine L. Gerst, Orlando Vargas Ramirez, Philip W. Rundel

**Affiliations:** 1 Department of Ecology and Evolutionary Biology, University of California, Los Angeles, CA 90095, USA; eriordan@ucla.edu (E.C.R.);; 2 Arizona-Sonora Desert Museum, Tucson, AZ 85743, USA; 3 La Selva Biological Station, Puerto Viejo de Sarapiquí 41001, Costa Rica; orlando.vargas@tropicalstudies.org

**Keywords:** Araceae, Cyclanthaceae, diversity, ecology, hemiepiphyte, Neotropics

## Abstract

Numerous plant functional traits of ecophysiology and morphology associated with an epiphytic life history have promoted relatively high rates of evolutionary diversification and ecological success in tropical families such as the Orchidaeae, Polypodiaceae, Bromeliaceae, and Cactaceae. Epiphytic life histories are relatively uncommon in the Araceae and rare in the Cyclanthaceae which lack key functional traits for epiphytism. Only two lineages of Neotropical Araceae, *Anthurium* and *Philodendron*, include examples of epiphyte life histories. The evolution of a hemiepiphytic life history represented an important development for tropical Araceae by providing functional traits that have greatly expanded opportunities for adaptive radiation and ecological success as indicated by species richness and frequency of occurrence. The key adaptive trait allowing the diversification of hemiepiphytic Araceae was the development of heteroblastic growth of leaves and stems. Although hemiepiphytic life histories are present in the Cyclanthaceae, the family has undergone only modest speciation and limited ecological success in both its epiphytes and hemiepiphytes. Extensive sampling of more than 4600 trees from primary forest on four soil groups in northeastern Costa Rica have found a modest diversity of 15 species of epiphytic Araceae but only two species of epiphytic Cyclanthaceae. In contrast, 38 species of hemiepiphytic Araceae and 5 species of hemiepiphytic Cyclanthaceae were sampled, indicating relatively limited adaptive radiation of hemiepiphytic Cyclanthaceae and lower ecological success. Using summed values of frequency of occurrence as a measure of ecological success, epiphytic Araceae were 18 to 42 times more frequent than epiphytic Cyclanthaceae in swamp, alluvial, and residual soil forests. Summed frequencies of occurrence of hemiepiphytic Araceae were 7 to 13 times higher than those of hemiepiphytic Cyclanthaceae. The four soil groups were similar in their floristic composition of epiphytic and hemiepiphytic Araceae and Cyclanthaceae, but the frequencies of occurrence of both epiphytes and hemiepiphytes were, with few exceptions, highest on swamp soil plots, with alluvial soil plots slightly less favorable.

## 1. Introduction

Members of the Araceae and Cyclanthaceae form important components of the understories of Neotropical rainforests where they share dominance with species of Costaceae, Heliconiaceae, Marantaceae, and Zingiberaceae [1]. In contrast to a terrestrial understory environment with direct root connection to the soil, epiphytic life histories have not widely evolved in many of these families [2]. Even in the wet and humid conditions of a rainforest environment the challenges for successful growth and survival of true epiphytes are significant for vascular plants. There are, however, families which provide classic examples of the evolution of epiphyte life histories with species richness and/or ecological success in Neotropical forests. These families include the Orchidaceae, Polypodiaceae, Bromeliaceae, and Cactaceae which each display adaptive functional traits in morphology, ecophysiology, and phenology to allow their survival in an epiphytic environment without a direct root connection to the soil [2,3]. These include morphological structures for water and nutrient impoundment [4], xeromorphism [5], resurrection capacity [6], Crassulacean acid metabolism [7,8], and ecophysiological traits that increase desiccation tolerance and microhabitat selection [6].

Without such functional traits there is little or no possibility of adaptive radiation from lineages of terrestrial understory herbs into the canopy of tropical forests. It is not surprising, therefore, that the Costaceae, Heliconiaceae, Marantaceae, and Zingiberaceae have not been successful in making any significant transition to epiphytic life histories. However, several lineages with a modest diversity of epiphytic species have evolved in the Araceae and Cyclanthaceae, both families which lack obvious adaptive traits for epiphytism. This evolution is best seen in scattered lineages of *Anthurium* and more modestly *Philodendron*. The Cyclanthaceae, which shows a convergence in plant growth forms with the Araceae, is very poor in epiphytes and minimal adaptive traits allowing an epiphytic life history [9].

In contrast to epiphytes, the hemiepiphytic life history was a major evolutionary development for the Araceae and Cyclanthaceae in providing ecophysiological, morphological, and anatomical traits that have greatly expanded the potential opportunities to not only survive their canopy habitats but undergo adaptive microhabitat radiation and achieve impressive ecological success. The evolution of a hemiepiphytic life history distinct from that of true epiphytes and tropical vines is unique to the Araceae and Cyclanthaceae [9].

The hemiepiphytic life history begins with seeds germinating on the forest floor and producing herbaceous stems which crawl across the forest floor. Upon encountering a suitable host trunk, the stems use attachment roots to climb vertically up the trunk [10]. This habitat transition from terrestrial to epiphytic is associated with the nodal production of two forms of aerial roots. Short, branched anchor roots attach the stem firmly to the host trunk, while long and unbranched feeder roots grow downward to reach the soil and establish new hydraulic plant–soil linkages [11,12,13]. Early in the developmental stage when the young plants have climbed to heights of 1–2 m there is a characteristic senescence of the base of the main stem, cutting off uptake of moisture from the rhizome [9,13,14,15,16].

Much of the diversity of Araceae reported in studies of tropical epiphyte communities lies with hemiepiphytic species rather than true epiphytes. As reviewed by Zotz [5], the term hemiepiphyte dates back more than a century to Fritz Went [17] who distinguished hemiepiphytes from true epiphytes via their production of aerial roots which reached to the soil. Schimper [18] provided a more specific definition by limiting the term to epiphytes that germinate in tree crowns and later establish root connections to the soil via aerial roots. He added the term pseudoepiphytes for species that germinate on the ground and later climb trees with a characteristic subsequent dieback of the proximal stem portion, describing what we term simply as hemiepiphytes. More recently, several authors proposed that ecologists distinguish primary hemiepiphytes that begin life as an epiphyte and later develop a ground connection through aerial roots, as with strangler figs, from secondary hemiepiphytes that were equivalent to Schimper’s pseudoepiphytes [18]. We suggest that the widely used definition of primary hemiepiphytes establishes an eclectic mix of species with unrelated adaptive traits linked only by their ability to establish a ground connection with aerial roots later in life. These species categorized as primary hemiepiphyte include strangler species of *Ficus* and *Clusia*, normally terrestrial woody species able to root in canopy soil pockets and establish aerial roots, some lianas such as *Marcravia*, and ferns growing low on tree trunks which develop aerial roots [19,20,21]. In our work we restricted use of the term hemiepiphyte to include only species in the Araceae and Cyclanthaceae with a unique life history and formerly termed secondary hemiepiphytes.

Our objectives in carrying out this research had two elements. The first of these was to compare and contrast the species richness and frequency of occurrence of epiphytic and hemiepiphytic members of the Araceae and Cyclanthaceae in a humid rainforest environment in Costa Rica. Most existing studies of Neotropical epiphyte communities have not distinguished between epiphytes and hemiepiphytes in presenting data on species richness and ecological frequency of occurrence [22,23,24,25,26]. The Araceae forms a large and diverse family with about 100 species at the La Selva Biological Station in northeastern Costa Rica. However, there are only a modest number of epiphytes and almost all species of *Anthurium.* An example of evolutionary radiation and ecological success in the family is much more apparent with hemiepiphytes. However, the Cyclanthaceae with seemingly similar adaptive traits of life history has not achieved either great species diversification or ecological radiation into specialized microhabitats [9]. The second objective has been to ask if the forest soil substrate impacted the community structure and relative abundance of epiphytes and/or hemiepiphytes. Using our field studies of epiphytes and hemiepiphytes in the Araceae and Cyclanthaceae, we compared and contrasted the epiphytic and hemiepiphytic floras on trees growing in four distinct soil groups (in order of frequency of occurrence at La Selva: residual, alluvial, riparian, and swamp soils). We hypothesized that hemiepiphytes with a root connection to the soil in the early stages of their life history should be favored in their establishment on swamp soils compared to epiphytes.

## 2. Materials and Methods

Field studies were conducted at the La Selva Biological Station, a 1600-hectare reserve of premontane wet forest in the Atlantic lowlands of northeastern Costa Rica (10°28′ N, 83°59′ W). The research station has a mean annual rainfall of 4244 mm (1958–2004), with mean monthly rainfall above 300 mm from May through December. There are peaks of precipitation above 400 mm mo^−1^ in June–August and November–December and a drier period from January through April. Even in the driest period of February and March, however, rainfall averages above 150 mm each month. Mean monthly maximum and minimum temperatures show little seasonal change, with mean highs of 30–31 °C each month and mean monthly lows ranging only from 20–22 °C. Research permits were granted by the “Comisión Institucional de Biodiversidad” (Institutional Biodiversity Committee, University of Costa Rica; resolution VI-8315-2014) and authorized by La Selva Biological Station. The identifications of species were confirmed using a large reference collection in the La Selva Herbarium [9]. Multiple voucher specimens for each study species are archived in the herbarium of the La Selva Biological Station (LSCR). This collection was founded in 1995 and contains more than 10,000 pressed plant specimens. Specific collection numbers are available from the authors. Species names used in this article follow Hammel et al. [27].

Field measurements were carried out within areas of old growth forest at La Selva representing four distinct edaphic conditions—residual soils, alluvial soils, riparian soils, and swamp soils. Residual soils form the most extensive soil type at La Selva and are present on rolling upland terrain where young volcanic flows have been weathered and leached. These residual soils are strongly acidic, rich in organic matter, and highly leached with a low degree of base saturation leached [28,29]. The canopy of these forests is heavily dominated by *Pentaclethra macroloba* (Fabaceae) which typically attain canopy heights of 30–35 m. Mixed with the *Pentaclethra* are taller canopy trees that reach heights of 40–55 m, giving the forest an irregular upper canopy structure. The tall subcanopy palms *Welfia regia*, *Iriartea deltoidea*, and *Socratea exorrhiza* are a distinctive component of these forests and reach large diameters of 30 cm or more [30]. The understories of these primary forests exhibit a rich diversity of species that flower and fruit successfully in the understory at heights of 5–15 m [30].

Alluvial soils occur on terraces bordering the major rivers at La Selva and especially along the Rio Sarapiquí. These are relatively flat Pleistocene terraces that are no longer flooded. The alluvial soils are fertile and base-rich with neutral pH values of about 6.0 soil pH [28,29]. *Pentaclethra macroloba* continues as a dominant canopy species but with the addition of *Dipteryx panamensis* (Fabaceae), *Hymenolobium mesoamericanum* (Fabaceae), and *Sloanea laevigata* (Elaeocarpaceae). Notably absent is *Carapa nicaraguensis* (Meliaceae). Characteristic understory species are *Preslianthus pittieri* (Capparaceae) and the colonial palm *Bactris coloradonis* [30].

Riparian forest vegetation is restricted to the margins of perennial streams in the valley bottoms of La Selva where soil water is continuously available. These soils are poorly to moderately drained, highly acidic, and poor in exchangeable bases [29]. Characteristic tree species in this habitat include *Ficus insipida* (Moraceae), *Zygia longifolia* (Fabaceae), *Cordia lucidula* (Boraginaceae)*, Inga marginata* (Fabaceae) *Nectandra reticulata* (Lauraceae), and *Posoqueria latifolia* (Rubiaceae). Other common canopy tree species that are shared with alluvial forest soils are *Inga ruizana* (Fabaceae), *Luehea seemannii* (Malvaceae), and *Myrcia splendens* (Myrtaceae) [30].

Swamp soils with primary forest occur on poorly drained areas where standing water 20–30 cm deep remains for 3–5 days or more after heavy rains [30]. These soils are clay-rich and extremely acidic with a pH of 4.0–4.2 [28,29]. *Pentaclethra* dominates these stands along with *Luehea seemannii* (Malvaceae), *Otoba novgranatensis* (Myristicaceae), *Pachira aquatica* (Malvaceae), and *Pterocarpus officinalis* (Fabaceae). A diverse set of subcanopy trees are present but few are primarily restricted to the swamp soils [30]. Exceptions are *Cojoba* sp. (Fabaceae) and *Grias cauliflora* (Lecythidaceae). Characteristic understory species include *Adelia triloba* (Euphorbiaceae), *Chione venosa* (Rubiaceae), and *Psychotria chagrensis* (Rubiaceae).

Fifteen sample sites were selected in 2005 within each of the residual, alluvial, riparian, and swamp soil groups (Supplemental Figure S1). One additional site was added for swamp soils to increase the number of trees sampled because of the lower tree density in these soils. Sites were randomly selected with restrictions to avoid ecotonal or disturbed area. Sites had to be at least 25 m from any trail and could not border between habitat types. At each site, a 10 m radius circular plot (314 m^2^) was surveyed for the presence of epiphytic and hemiepiphytic species of Araceae and Cyclanthaceae. The one exception was the decision to not sample *Monstera* because of the difficulty of identifying juvenile growth forms. Ten species of this genus, all hemiepiphytes, are reported for La Selva. All trees above 2.5 cm diameter at breast height (dbh) were measured and the presence of hemiepiphytes or epiphytes was recorded. The number of trees sampled for each of the four groups of soil plots varied as density differed between plots. In total, 4586 trees were surveyed in the course of this study as follows for the four soil groups—residual (1162 trees), alluvial (887 trees), riparian (1691 trees), and swamp (848 trees) (see Supplemental Data File S1).

We use the term hemiepiphyte in this study to distinguish root-climbing species of the Araceae and Cyclanthaceae from true epiphytes and climbers in other families [9]. Thus, we include only species within these two families with a specific life history that begins with a germinated seed on the forest floor, linear growth until a tree is encountered, and development of aerial attachment roots, eventual senescence of basal stem, and replacement of hydraulic connection to the soil with another group of aerial roots. Some researchers have suggested that secondary hemiepiphytes do not form a distinct growth form with adaptive traits distinct from other vine species and would be better termed nomadic vines [5,11,31,32,33]. We have argued that this is a poor choice of name for a variety of reasons and that there are specific life history traits that distinguish a group we consider as hemiepiphytes that are only present in the Araceae and Cyclanthaceae [9].

We tested for differences in community composition based on soil condition as a grouping factor using an analysis of similarity (ANOSIM)with the Bray–Curtis distance metric to quantify the dissimilarity between and within groups. An R statistic compared the mean of ranked dissimilarities between groups to the mean of ranked dissimilarities within groups. An R value close to “1.0” suggests dissimilarity between groups while an R value close to “0” suggests an even distribution of high and low ranks within and between groups. Community composition for the test was a matrix of species abundance at each plot (15 plots per soil type and 60 plots total) for all aroids and cyclanths including both epiphytes and hemiepiphytes.

## 3. Results

### 3.1. Soil Substrate

**Figure 1:**
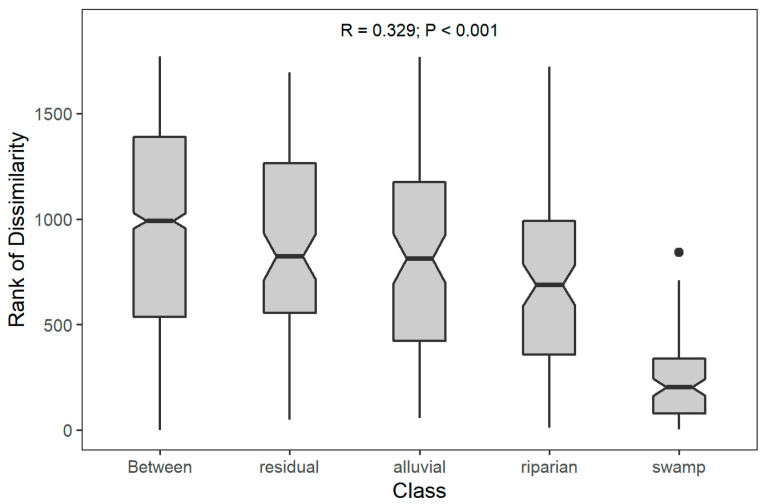
Analysis of similarity (ANOSIM) plot to test for dissimilarity in community composition based on soil condition as a grouping factor showing that the swamp soil group is distinctive from the other groups and from the other soil groups together.

An analysis of similarity (ANOSIM) plot was used to test for dissimilarity in community composition of epiphytic and hemiepiphytic Araceae and Cyclanthaceae based on forest soil as a grouping factor. Community composition using a matrix of species abundance at each plot (15 plots per soil type and 60 plots total) showed that epiphyte and hemiepiphyte communities on swamp group soils were statistically distinctive from those on other soil groups (r = 0.329, *p* < 0.001); (Figure 1).

Epiphytic and cyclanths and aroids had their greatest abundance in swamp soil plots, which was typically 30 to 500% higher compared to other soil types (Table 1). The summed frequency of occurrence for cyclanths epiphytes was very low ranging from 0.5 to 3.3 for the four soil groups, compared to sums of 33.3 to 67.8 for aroid epiphytes (Table 1). The ratio of aroid epiphyte species to cyclanth epiphyte species ranged from 7.8 to 8.5 for the four soil groups (Table 1). For both families, hemieiphytes had a notably greater frequency of occurrence. Among the 37 aroid hemiepiphyte species sampled, 24 had their highest frequency of occurrence in the swamp forest plots, another six had the highest frequencies in alluvial soil plots, five species in alluvial plots and two in riparian plots. 

### 3.2. Epiphytes

**Figure 2:**
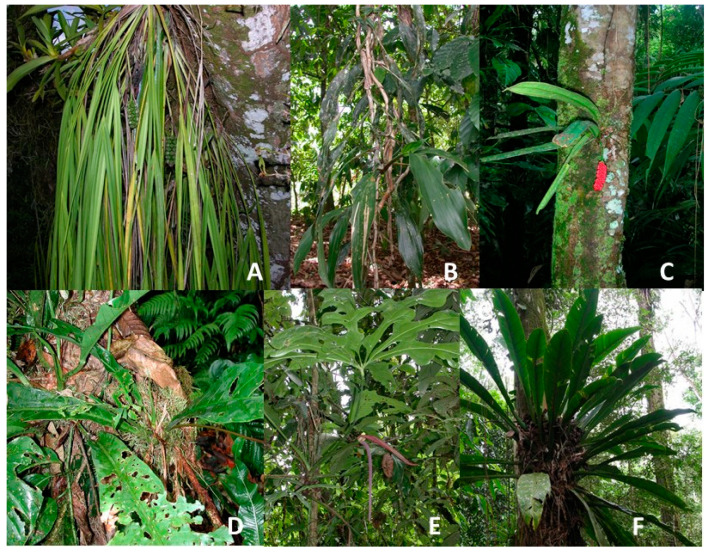
Epiphytic species of Cyclanthaceae and Araceae at the La Selva Biological Station: (**A**) *Chlorigyne pendula*, Cyclanthaceae; (**B**) *Ludova integrifolia*, Cyclanthaceae; (**C**) *Anthurium bakeri*, Araceae; (**D**) *Anthurium consobrinum*, Araceae; (**E**) *Anthurium clavigerum*, Araceae; (**F**) *Philodendron wendlandii*, Araceae. Photos (**A**,**F**) are by O. Vargas; photos (**B**,**D**,**E**) by R. Aguilar; and photo (**C**) by R. Sabicetti.

True epiphytes which lack a hydrologic connection to the soil are few in number in the Cyclanthaceae [9]. This can be seen clearly in the relative rarity of epiphytic Cyclanthaceae present in the sample plots. *Ludovia integrifolia* (Figure 2B) was uncommon and *Chlorigyne pendula* (Figure 2A) had a frequency of 2.3% of trees in the swamp plots, and low levels in the other three soil plots (Table 2). The genus *Sphaeradenia* has two rare species at La Selva, only one of which was found in our plots, occurring on only one tree of the over 4500 surveyed.

In contrast, our field samples recorded sixteen epiphytic species of Araceae, with fourteen species of *Anthurium* and two species of *Philodendron* (Table 2). *Anthurium consobrinum* (Figure 2D) was the overwhelmingly common aroid epiphyte encountered in the samples from all four soil types. This species was extremely common in swamp, alluvial, and residual plot trees, occurring in 19.6% to 22.5% of trees samples, with a lower but still impressive frequency of occurrence of 11.3% on trees in riparian soil plots. *Anthurium bakeri* (Figure 2C) was also common, occurring on 17.9% of trees in swamp sites and 6.0% to 10.2% in other soils. Ten of the sixteen epiphytic species of aroids had a higher frequency of occurrence on trees in the swamp plots than in the other three soil types.

Twelve of the aroid epiphyte species were present in the sample plots for all four soil types (Table 2). Eight epiphytic species of Araceae had a frequency of occurrence of 1% or more in the swamp plots. These, in order of frequency of occurrence, were *Anthurium consobrinum* (22.5%; Figure 2D), *A. bakeri* (17.9%; Figure 2C), *A. ramonense* (5.7%), *A. cuspidatum* (4.6%), *A. clavigerum* (4.5%; Figure 2E), and *A. upalense* (3.6%), *Philodendron radiatum* (3.4%), and *P. wendlandii* (3.4%). 

### 3.3. Hemiepiphytes

**Figure 3:**
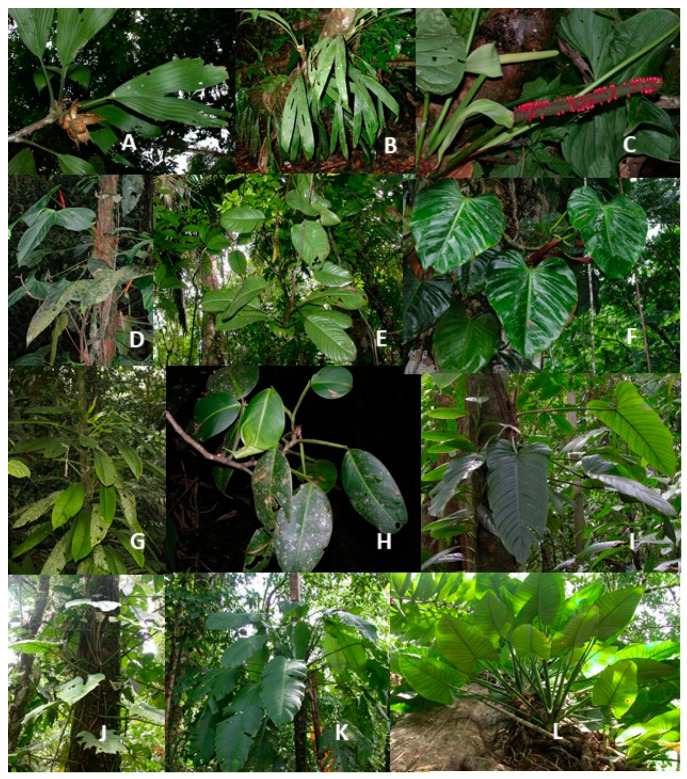
Hemiepiphytic species of Cyclanthaceae and Araceae at the La Selva Biological Station: (**A**) *Asplundia utilis*, Cyclanthaceae; (**B**) *Evodianthus funifer*, Cyclanthaceae; (**C**) *Anthurium formosum*, Araceae; (**D**) *Anthurium subsignatum*, Araceae; (**E**) *Philodendron alliodorum*, Araceae; (**F**) *Philodendron platypetiolatum*, Araceae; (**G**) *Philodendron rhodoaxis*, Araceae; (**H**) *Philodendron rigidifolium*, Araceae; (**I**) *Philodendron jodavisianum*, Araceae; (**J**) *Philodendron brunneicaule*, Araceae; (**K**) *Rhodospatha wendlandii*, Araceae; (**L**) *Syngonium schottianum*, Araceae. Photos (**A**,**J**) are by E. Riordan; photos (**B**,**C**,**F**–**I**) by O. Vargas; and photos (**D**,**E**,**K**,**L**) are by R. Aguilar.

Although only six species of hemiepiphytic Cyclanthaceae were present at La Selva, two high climbing species were quite abundant. *Asplundia utilis* had its highest frequency of occurrence of 8.8% on alluvial soils, dropping to 6.3% on swamp forest soils (Figure 3A). Three other species of *Asplundia* were encountered with *A. ferruginea* reaching a frequency of 2.8% on trees in both the riparianand residual soil plots. *Asplundia euryspatha* and *A*. *multistaminata* had frequencies at or below 1% of the trees in any group of plots. *Evodianthus funifer* (Figure 3B) had a high frequency of occurrence of 9.6% in swamp forest plots and 7.0% in alluvial soil plots (Table 3). Its frequencies were lower at 2.0 and 5.7% in residual and riparian soil plots.

A rich diversity of aroid hemiepiphytes was present in our sample plots with thirty-seven species distributed across *Anthurium* (six species), *Philodendron* (twenty-three species), *Rhodospatha* (two species), and *Syngonium* (six species). Not included in our sampling regime were species of *Monstera* because of uncertainties with identification. The number of species present in our samples from each soil group ranged from 34–31 species of Araceae, but only 26 aroid species were present in our sample plots from all four soil types (Table 3).

The diversity of hemiepiphytic species of *Anthurium* was lower than that of epiphytic species of this genus in our plots. Hemiepiphytic species of *Anthurium* showed a similar pattern of distribution favoring swamp soil plots but lacking were high frequencies of occurrence compared to those of other genera of Araceae. (Table 3). *Anthurium formosum* (Figure 3C) and *A. subsignatum* (Figure 3D) had the highest frequencies of occurrence. The swamp soil plots generally were found to have the highest frequencies of *Anthurium* species occurrence with up to 3–6% and the lowest frequency in residual soil plots of 1.0% or less. The one exception to this pattern was observed in *A. pentaphyllum* with its highest but relatively modest frequency of occurrence of 2.7% in residual soil plots.

Higher frequencies of occurrence were present in other aroid genera. Three quarters of the aroid hemiepiphyte species were present on 1% or more of the trees in one or more of our four groups of soil plots. Fourteen species reached frequency levels above 5% in one or more of the soil groups. Comparing individual species, *Philodendron alliodorum* (Figure 3E) was the most abundant hemiepiphytic aroid in each of the four soil groups and showed remarkable ecological success in its presence on 55.8% of the sampled trees in the swamp forest (Table 3). In the alluvial forest it was sampled on 32.8% of trees and still an impressive 26.3% of the trees on residual soil plots.

Next common in frequency of occurrence among hemiepiphytic *Philodendron* species was *P. platypetiolatum* (Figure 3F) with a frequency of 15.4% of trees on swamp forest soils and 11.3% of trees in our riparian soil plots (Table 3). It was present in the alluvial and residual soil plots with lower but still significant frequencies of 7.9 and 4.0%, respectively. This species favors open-light environments and grows loosely attached to understory or canopy trees.

Many other hemiepiphytes showed extraordinary abundance with a presence on more than 10% of sampled trees in one group of soil plots (Table 3) parallel to the observations of other researchers who have consistently reported high species diversity and ecological success of hemiepiphytic aroids [26,27,28,29]. Additional species of *Philodendron* with a high frequency of included *P. rhodoaxis* (Figure 3G), a mid-canopy climber, showed the common pattern of having its highest frequency on trees (10.2%) in the swamp forest plots and 5.1% on trees in alluvial plots. This pattern was also found for *P. jodavisianum* (Figure 3I), *P*. *tripartitum,* and *P. herbaceum*.

*Philodendron rigidifolium* (Figure 3H) is an unusual species with masses of aerial roots and similarly had a high frequency of 10.1% in residual plots and was uncommon in plots of other soil types (Table 3). This unusual climber reaches high into the canopy where it actively branches and produces enormous numbers of pendant aerial roots that drop to the forest floor where they root. *Philodendron brunneicaule* (Figure 3J) had a modest frequency of occurrence in swamp plots but was only rarely present on plots with other soil types.

Extremely common in frequency of abundance and frequency of occurrence was *Rhodospatha wendlandii* (Figure 3K) which was present on 32.0% of trees in swamp forest plot and high as well as 22.2% and 18.9% of trees in riparian and alluvial soil plots, respectively (Table 3). The frequency of occurrence was somewhat lower on trees in the residual soils at 9.7%. This thick-stemmed climber reaches up to 30 m in the canopy where it occurs abundantly. Juvenile plants were common compared to other species. Much more specialized in its habitat was the climber *Philodendron fragrantissimum* with a frequency of 10.4% in residual soil plots, but it was absent from the other three soil types (Table 3).

Another species with a variable abundance between soil groups was *Syngonium triphyllum* with a frequency of occurrence 17.0% in the swamp soil plots (Table 3). Its frequency of occurrence on riparian plot soils was 10.4. The frequencies of *S. triphyllum* dropped to 2.7% and 8.3% on residual and alluvial soils, respectively. This climber reaches middle canopy positions from which long flagellar stem segments frequently become pendant and are dropped to the soil or bridge the gaps between tree canopies. *Syngonium macrophyllim* and *S. schottianum* (Figure 3L) each displayed a pattern of relatively high frequencies of occurrence in swamp, alluvial, and riparian soil plots and lower frequencies in residual soil plots.

## 4. Discussion

### 4.1. Epiphytes

The Cyclanthaceae and Araceae have had only limited success in evolving the critical adaptive traits to allow their success as true epiphytes as has occurred in the evolutionary diversification of epiphytic Orchidaceae or abundance and ecological success of epiphytic Bromeliaceae and Polypodiaceae. The low diversity and ecological abundance of both epiphytic cyclanths and epiphytic aroids compared to the larger number of species and greater ecological abundance of aroid and cyclanths to hemiepiphyte species (Table 1) speaks to the challenges of finding favorable microhabitats with sufficient water resources to allow survival and reproduction.

The three genera of epiphytic Cyclanthaceae found in our sampling show no obvious specialization for an epiphytic life history. *Chlorigyne pendula* is a high canopy species with a branched stem and pendulous leaf display. Large root mats may provide moisture storage for mature individuals, but it is not clear how small plants obtain their supply of water. *Ludovia integrifolia* is a poorly studied species which takes on a branched liana-like growth form that can extend for 10–20 m or more through the canopy. Despite this large size we have not been able to find any direct hydraulic connection to the soil with aerial roots in this species. The third genus, *Sphaeradenia* species typically have a short unbranched stem up to 1 m in length with a combination of short aerial roots for attachment and for moisture uptake [9]. Both *Chlorogyne* and *Sphaeradenia* are occasionally found surviving at ground level along trails in the forests after branch falls, suggesting that they have a sufficient root mass to store moisture. Epiphytic Cyclanthaceae are limited to three genera which lack obvious adaptive traits for an epiphytic existence. *Ludovia*, a widespread cyclanths genus with three species, deserves careful study. Although commonly described as an epiphyte, *Ludovia* typically grows as a liana with an extensive length extending within tree canopies. 

Aroid epiphytes in Neotropical forests occur only in two genera, *Anthurium* and *Philodendron*, and are virtually absent in Paleotropical forests. The frequencies of occurrence of the aroid epiphytes are relatively low compared to those of many hemiepiphytic aroids. With one exception, the frequency of occurrence for the most successful aroid epiphytes was a presence on 3–5% of trees, with their highest frequencies in swamp forests where humidity is high. The notable exception to this pattern occurs with *Anthurium consobrinum and A. bakeri*, which were present on nearly 20% or more of trees in the swamp forest plots. A high frequency of occurrence for *A. bakeri* has been noted in the past in primary forest on residual soils at La Selva [34].

The majority of epiphytic aroids at La Selva are small plants, usually more or less acaulescent or caespitose, with a short coarse stem. Typically, there are two forms of aerial roots, with one specialized for attachment and the other forming thick mats for moisture uptake from the bark or arboreal pockets of branch soil [34]. This group includes many representatives from the Sections *Pachyneurium* and *Porphyrochitonium*. Included here are *A. bakeri*, *A. acutiangulum*, *A. Austin-smithii*, *A. bradeanum*, *A. llanense*, *A. friedrichsthallii*, *A. gracile*, *A. obtusum*, *A. ramonense*, and *A. spathiphyllum* from La Selva [35].

Three epiphytic species of aroid at La Selva have evolved a rosulate growth form which could function as a pseudo-tank for water storage in the manner that many epiphytic Bromeliaceae utilize tanks to store rainwater [4]. *Anthurium consobrinum* (Figure 2D), *A. upalense*, and *Philodendron wendlandii* (Figure 2F) with this growth form might be expected to have a favorable advantage over species lacking this morphology [34]. The two *Anthurium* species may receive some benefit from their rosulate growth form as they showed moderate but not exceptional frequencies of occurrence. *Philodendron wendlandii*, however, was relatively rare and did not appear to benefit in obvious ways.

Two species included here as epiphytes have an unusual life history trait that has led them to be lumped as primary hemiepiphytes by some authors [36]. They begin their lives as epiphytes but with age develop aerial roots that eventually reach the soil and establish hydrologic pathways for water and nutrient uptake *Philodendron radiatum* generally becomes established on relatively high branches as much as 30 m above ground level. These plants with maturity develop pendant aerial roots that extend downward to connect with the soil, eventually becoming stranglers. Small plants lower in the shaded canopy appear to survive poorly and do not develop a soil connection. 

*Anthurium clavigerum* is a robust species that in contrast becomes established low in the canopy along forest edges [36]. This species begins life as an epiphyte in the lower canopy, usually no more than a few meters above the soil. As it matures it sends aerial roots toward the ground and develops firm soil connections for water and nutrient uptake.

### 4.2. Hemiepiphytes

In contrast with the limited diversity and small ecological role played by epiphytic Araceae, the species richness and ecological abundance of hemiepiphytes is high. *Philodendron alliodorum* (Figure 3E) was typically the most common aroid hemiepiphyte in the primary forest, particularly in the lower canopy throughout La Selva. The primary stem connection is lost at an early stage of growth connection and replaced by secondary aerial roots. However, small plants may spend some time surviving on moisture and nutrient uptake from their attachment roots. As a young plant develops it extends rapidly up the host trunk branch to the mid to high canopy. Once it reaches this height it exhibits repeated branching and accumulates a large amount of canopy biomass. Pendant stems may descend to the ground. Often what appear to be young stems on the forest floor are vegetative branches that are interconnected with other pendent stems of adult canopy plants or one that has climbed up a tree and sent down pendent stems to the ground. Fragmentation of stems of pendant stems by wind or fallen branches often causes asexual establishment and renewed growth of stem fragments.

As with *Philodendron alliodorum*, we commonly observed small branch sections of the common *P. platypetiolatum* (Figure 3F) that had broken off from a main stem and reestablished themselves low on the trunk without any obvious secondary roots attached to the soil. It is rare to see plants connected to soil with their primary root, even when small. Vegetative growth up a host tree is rapid and plants reach the mid to high canopy level where they branch extensively and develop large biomass. At least five of the hemiepiphytic *Philodendron* species at La Selva have a complex life history in that they begin life in the soil and climb trees but as they mature produce aerial roots that descend to soil level where they root. The most notable of these is *P. rigidifolium* (Figure 3H) which produces hundreds of pendulous roots in large masses that reach the soil.

Complex developmental patterns of heteroblastic growth are a critical element to the ecological success of many vines and most hemiepiphytes [37,38]. Classic examples of this developmental flexibility have been described for *Syngonium tryphyllum* and *Philodendron fragrantissimum* [39,40,41]. When these species overgrow a supporting tree, the stem responds to the loss of contact with the tree by switching to a new developmental pathway, forming thin flagellar stem segments with reduced leaf area. As these shoots descend to the ground level they grow laterally across the soil surface until they contact another tree. At this point the stems switch from flagellar growth to a morphology with short internode and thicker stems with increased leaf area determine the shoot’s position along a continuum from sessile to mobile. Most individual shoots show little change in shape as successive segments. The size and shape of the internodes and the pattern by which they change along the shoot determine the shoot’s position along a continuum allows the plant great mobility for the colonization of neighboring trees [39,40,41].

Heteroblasty has also been an important adaptive trait that has promoted weedy growth in a few aroid hemiepiphytes and making them invasive species of concern. Rapid expansion of range facilitated by weedy growth is exemplified by *Epipremnum pinnata*, originally from the western Pacific which is widely invasive, forming masses of hanging stems in wet tropical forests as in in Sri Lanka and Hawai’i [42]. *Monstera deliciosa* and *Philodendron giganteum* are other invasive hemiepiphytes of concern [43].

The heights reached by hemiepiphytic aroids into the more exposed upper canopy levels have been shown to be associated with their ability to utilize their flexible growth phenologies to find and exploit microhabitats of branching architecture and light regime rather than stochastic processes [37,38]. Patterns of niche selection can be attributed to morphological and ecophysiological traits of the species including fine tuning of anatomical and ecophysiological traits that increase leaf succulence, sclerophylly, and epidermal resistance to water loss [44,45,46].

The association of species richness and ecological radiation with the evolution of heteroblasty in leaf and stem phenology of hemiepiphytic Araceae is important in comparisons of the two families [9]. The remarkable convergent development of many adaptive functional traits between the hemiepiphytic Cyclanthaceae and Araceae is not complete as heteroblasty has not been reported for the Cyclanthaceae.

## 5. Conclusions

Neither the Araceae or Cyclanthaceae have demonstrated great diversity or ecological success in evolutionary radiation as true epiphytes compared to other families with more obvious adaptive traits for an epiphytic life history. In contrast, the unique life history of hemiepiphytic growth has evolved successfully in both families although with far greater diversity and ecological impact in the Araceae. The key adaptive trait allowing for great diversification and ecological impact has been the development of heteroblasty in leaves and stems of hemiepiphytic Araceae, allowing greater adaptation to canopy microhabitats. The epiphyte and hemiepiphyte communities on trees in swamp soils differed significantly from that on other soil groups.

## Figures and Tables

**Table 1:** Species richness and summed frequencies of occurrence of epiphytic and hemiepiphytic Araceae and Cyclanthaceae in forest plots for four distinct soil units at La Selva Biological Station.

	Residual	Alluvial	Riparian	Swamp
Epiphytes				
Araceae				
Species	15	14	13	14
Summed frequencies	35.89	50.28	33.25	67.75
Cyclanthaceae				
Species	2	2	3	2
Summed frequencies	0.6	0.79	0.52	3.31
Ratios Araceae to Cyclanthaceae				
Species	7.5	6.5	6	6.5
Summed frequencies	59.8	63.6	63.9	20.5
Hemiepiphytes				
Araceae				
Species	31	32	32	34
Summed frequencies	101.19	130.97	140.85	212.52
Cyclanthaceae				
Species	4	4	4	4
Summed frequencies	8.26	18.04	15.6	17.13
Ratios Araceae to Cyclanthaceae				
Species	7.8	8	8	8.5
Summed frequencies	12.3	7.3	9.0	12.4

**Table 2:** Frequency of occurrence on trees for epiphytes in the Araceae and Cyclanthaceae from forest plots in four distinct soil units at La Selva Biological Station.

	Residual	Alluvial	Riparian	Swamp
Araceae—Epiphytes				
*Anthurium acutangulum*	0.34	1.01	0.26	0.12
*A. austin-smithii*	0.86	0.34	0.77	0.24
*A. bakeri*	6.02	10.15	10.18	17.85
*A. bradeanum*	0.09	0.34	0.26	0.24
*A. clavigerum*	0.95	3.83	0.77	4.49
*A. consobrinum*	19.62	20.52	11.34	22.46
*A. cuspidatum*	0.43	1.92	3.74	4.61
*A. friedrichsthalii*	0.52	0.11	0	0.24
*A. gracile*	0.09	0	0	0
*A. llanense*	0.77	0.56	0.13	0
*A. obtusum*	0	0	0	0.24
*A. ramonense*	2.32	4.74	2.19	5.67
*A. spathiphyllum*	0.95	0.68	0.52	1.18
*A. upalaense*	1.03	1.69	0.77	3.55
*Philodendron radiatum*	1.38	3.04	1.29	3.43
*P. wendlandii*	0.52	1.35	1.03	3.43
Total	35.89	50.28	33.25	67.75
Cyclanthaceae—Epiphytes				
*Chorigyne pendula*	0.43	0.56	0.26	2.25
*Ludovia integrifolia*	0.17	0.23	0.13	1.06
*Sphaeradenia acutitepala*	0	0	0.13	0
Total	0.6	0.79	0.52	3.31

**Table 3:** Frequency of occurrence on trees for hemiepiphytes in the Araceae and Cyclanthaceae from forest plots in four distinct soil groups at La Selva Biological Station.

	Residual	Alluvial	Riparian	Swamp
Araceae—Hemipiphytes				
* Anthurium flexile*	0	0.11	0	0
* A. formosum*	1.03	0.79	2.58	6.26
* A. interruptum*	0.86	2.59	1.03	2.6
* A. pentaphyllum*	2.67	1.92	1.55	0.35
* A. subsignatum*	0.43	1.01	1.03	4.26
* A. trisectum*	0.86	0.11	6.83	3.55
* Philodendron alliodorum*	26.25	32.81	38.14	55.79
* P. aromaticum*	2.75	3.27	2.58	4.26
* P. cretosum*	0	2.14	1.29	5.67
* P. brevispathum*	0	0	0	0.12
* P. brunneicaule*	0	0.11	0.9	0.47
* P. cretosum*	0.77	1.47	3.22	1.54
* P. davidsonii*	0.34	0.11	0.39	1.18
* P. fragrantissimum*	10.41	0	0	0
* P. hederaceum*	0.09	0.34	0.26	0.59
* P. herbaceum*	1.72	6.99	5.15	5.44
* P. inaequilaterum*	4.48	9.13	3.22	5.08
* P. jodavisianum*	7.57	7.33	4.12	8.27
* P. ligulatum*	0.17	0	0.26	0.47
* P. opacum*	0.17	0	1.16	1.89
* P. platypetiolatum*	4.04	7.89	11.34	15.37
* P. rhodoaxis*	0.52	5.07	5.28	10.17
* P. rigidifolium*	10.07	2.03	2.19	1.42
* P. rothschuhianum*	0.26	0.45	0	0
* P. sagittifolium*	0.86	0.79	0.64	1.18
* P. schottii*	5.85	3.49	4.25	2.96
* P. sulcatum*	0.34	0.9	0.52	3.55
* P. tenue*	0	0.45	0.13	1.18
* P. tripartitum*	2.75	5.52	4.64	6.26
* Rhodospatha pellucida*	0.17	0.11	0.52	4.14
* R. wendlandii*	9.72	18.94	22.16	32.03
* Syngonium hoffmannii*	0.52	1.01	0.26	1.42
* S. macrophyllum*	1.64	3.27	3.22	2.36
* S. peliocladum*	0	1.35	0.13	1.18
* S. rayi*	0.52	0	0	0.12
* S. schottianum*	0.69	1.24	1.29	4.37
* S. triphyllum*	2.67	8.23	10.57	17.02
Total	101.19	130.97	140.85	212.52
Cyclanthaceae—Epiphytes				
* Asplundia euryspatha*	0	0.23	1.29	0.12
* A. ferruginea*	2.84	1.24	2.84	0.83
* A. multistaminata*	0.77	0.79	1.29	0.35
* A. utilis*	2.67	8.79	4.51	6.26
* Evodianthus funifer*	1.98	6.99	5.67	9.57
Total	8.26	18.04	15.6	17.13

## Data Availability

Data is contained within the Supplementary Material.

## Supplementary Materials

The following supporting information can be downloaded at: https://www.mdpi.com/article/10.3390/plants12234004/s1. File S1: Spreadsheets of field sample data for epiphytes and hemiepiphytes on residual, alluvial, riparian, and swamp soil stands. Figure S1: La Selva Biological Station soils map with plot locations. Plot locations indicated by circles (swamp), riparian (square), residual uplands (square), and alluvial lowland (triangle).
