# Supplementary material for: Differential Species Richness and Ecological Success of Epiphytes and Hemiepiphytes of Neotropical Araceae and Cyclanthaceae
Source: Plants (Basel). 2023 Nov 28;12(23):4004. doi: 10.3390/plants12234004 (PMC10708273; doi:10.3390/plants12234004)
Supplement: Supplementary file 1 [file plants-12-04004-s001.zip › plants-2646211-Supplementary Figure S1.pdf]

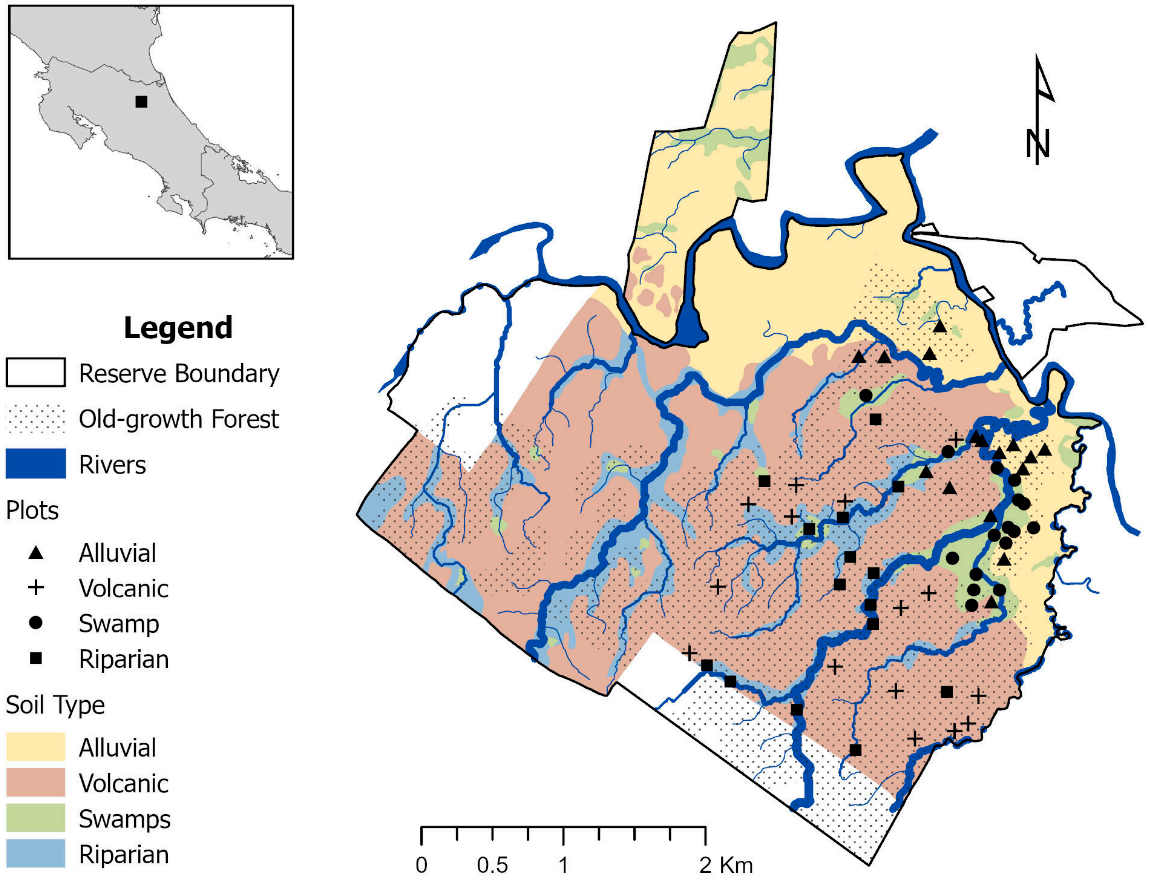

Figure S1: La Selva Biological Station soils map with plot locations. Plot locations indicated by circles (swamp), riparian (square), residual uplands (square), and alluvial lowland (triangle).
